# IGF-1 and Glucocorticoid Receptors Are Potential Target Proteins for the NGF-Mimic Effect of *β*-Cyclocitral from *Lavandula angustifolia* Mill. in PC12 Cells

**DOI:** 10.3390/ijms25189763

**Published:** 2024-09-10

**Authors:** Chenyue An, Lijuan Gao, Lan Xiang, Jianhua Qi

**Affiliations:** College of Pharmaceutical Sciences, Zhejiang University, 866 Yu Hang Tang Road, Hangzhou 310058, China; anchenyue@zju.edu.cn (C.A.); k923146@zju.edu.cn (L.G.)

**Keywords:** nerve growth factor, Lavender, *β*-Cyclocitral, neurogenesis, IGF-1 receptor, glucocorticoid receptor

## Abstract

In the present study, the PC12 cells as a bioassay system were used to screen the small molecules with nerve growth factor (NGF)- mimic effect from Lavandula angustifolia Mill. The *β*-Cyclocitral (*β*-cyc) as an active compound was discovered, and its chemical structure was also determined. Furthermore, we focused on the bioactive and action mechanism of this compound to do an intensive study with specific protein inhibitors and Western blotting analysis. The *β*-cyc had novel NGF-mimic and NGF-enhancer effects on PC12 cells, while the insulin-like growth factor-1 receptor (IGF-1R)/phosphatidylinositol 3 kinase, (PI3K)/serine/threonine-protein kinase (AKT), and glucocorticoid receptor (GR)/phospholipase C (PLC)/protein kinase C (PKC) signaling pathways were involved in the bioactivity of *β*-cyc. In addition, the important role of the rat sarcoma (Ras)/protooncogene serine-threonine protein kinase (Raf) signaling pathway was observed, although it was independent of tyrosine kinase (Trk) receptors. Moreover, the non-label target protein discovery techniques, such as the cellular thermal shift assay (CETSA) and drug affinity responsive target stability (DARTS), were utilized to make predictions of its target protein. The stability of IGF-R and GR, proteins for temperature and protease, was dose-dependently increased after treatment of *β*-cyc compared with control groups, respectively. These findings indicated that *β*-cyc promoted the neuron differentiation of PC12 cells via targeting IGF-1R and GR and modification of downstream signaling pathways.

## 1. Introduction

The rapid aging of the worldwide population is projected to lead to a significant increase in the percentage of individuals aged 65 and above, from 10% in 2022 to 16% by 2050. This demographic shift is expected to increase the costs associated with dementia from USD1.3 trillion in 2019 to USD2.8 trillion by 2030 [1]. Diseases associated with aging, including Alzheimer’s disease (AD), cancer, and diabetes, present increasingly complex challenges. AD, a prominent form of senile dementia, requires early detection, and recent advancements in blood biomarkers such as plasma p-Tau231 and p-Tau217 have shown potential for correlating with A*β* deposition and cognitive decline [2]. However, our understanding of the molecular mechanisms of AD, including cholinergic effects, A*β* amyloid deposition, oxidative stress, Tau protein hyperphosphorylation, and NMDA receptor signaling, is still incomplete. Currently, the primary treatments available drugs for AD contain tacrine, rivastigmine, galantamine, donepezil, huperzine A, and memantine [3], and investigational drugs such as Lecanemab [4] and Donanemab [5] also show promise in clinical trials. However, existing small-molecule drugs do not effectively halt the onset or reverse the progression of AD; the high treatment costs of antibody-based drugs limit their utilization.

Neurite outgrowth is fundamental to establishing neural circuitry throughout development and in the process of regeneration [6]. The self-renewal potential of central and peripheral axons is influenced by a variety of factors, including ribosomal location, proteomic profile, microtubule stability, and signaling pathways. Following nerve injury, molecular pathways such as MAPK, AKT/mTORC1/p70S6K, PI3K/AKT, BDNF/Trk, and Ras/ERK are activated and play a crucial role in axonal regeneration. Furthermore, astrocytic modulation, growth factors, and microRNAs contribute to this regenerative process [7]. In particular, neurotrophins, such as nerve growth factor (NGF) and brain-derived neurotrophic factor (BDNF), are crucial for neurite outgrowth and survival. It is imperative to understand the regulation of neurite growth, as the progressive loss of neuronal connectivity is a hallmark of AD pathology. Consequently, the study of neurite growth regulation has become a focal point in the field of AD research.

Nerve growth factor (NGF) plays a pivotal role in cognitive function, supporting the survival, development, and continual maintenance of neurons. Due to its significance in the development of AD through its interaction with the A*β* production mechanism [8] and cholinergic system [9], NGF has emerged as a potential target for drug development. However, the strong polarity and substantial molecular weight of NGF hinder its ability to traverse the blood-brain barrier [10]. As a result, current research is concentrating on strategies to deliver NGF into the brain. One highly studied approach involves the use of adeno-associated virus vectors expressing human NGF, specifically CERE-110. Although these vectors have been extensively investigated, further validation is required to determine their efficacy [11]. This limitation necessitates the exploration of additional small molecule modulators of NGF and its receptors, which can mimic or enhance neurotrophic effects. LM11A-31, a small molecule agonist that targets the p75 receptor of NGF, has been shown to reduce microglial activation in mouse models of AD and is currently undergoing clinical trials [12]. Furthermore, several other small molecules with NGF-mimic properties have been discovered by our group using traditional Chinese medicines such as AMA, Lindersin B, and Cucurbitacin B [13,14,15]. These discoveries were made through the utilization of the pheochromocytoma cells (PC12) bioassay system, a well-established model for studying neuronal differentiation and the molecular mechanisms of NGF action [16].

To discover the small molecules with NGF-mimic effect from natural products, the large screening for natural products was first performed in the early research. The extract of Lavandula angustifolia Mill, which is well known for antioxidant, anti-inflammatory, antibacterial, hypnotic, and memory-enhancing properties [17], was found to have a novel NGF-mimic effect on PC12 cells. To understand the active components of Lavandula angustifolia Mill, the isolation and purification of the extract of this plant was performed under the guide of a PC12 cell as a bioassay system. The *β*-cyclocitral (*β*-cyc), as its main component, was discovered and determined the chemical structure. This compound has been indicated that it has antifungal, antibacterial, and pesticidal properties [18]. However, there is currently scarce research on the impact of *β*-cyc on the ability to promote neurite outgrowth. In the present study, *β*-cyc demonstrated excellent NGF-mimic and NGF-enhancing properties in a PC12 cell bioassay system. To fully understand the underlying mechanisms of action and identify target proteins, critical steps must be taken to consider *β*-cyc as a potential candidate for further research and development.

Growth factors, such as NGF and insulin-like growth factor-1 (IGF-1), play a positive role in neurogenesis in the hippocampus [19]. IGF-1, in particular, plays a key role in regulating cognitive function. Upon binding with IGF-1, the IGF-1 receptor (IGF-1R) triggers the phosphorylation of its tyrosine kinase domain, initiating intracellular signaling that modulates cell growth, differentiation, and the various life activities of higher organisms [19]. Disruption of the PI3K/AKT/mTOR pathway in the brains of AD patients, which is associated with disease severity [20,21], underscores the importance of balancing IGF-1R/PI3K/AKT for AD treatment. Glucocorticoids have also been found to be involved in cellular proliferation, neurotransmitter synthesis, neuronal survival, and neuronal differentiation. These actions are closely linked to neurite outgrowth in PC12 cells [22]. Additionally, glucocorticoid receptors (GR) have been implicated in stress response and central nervous system functions, including learning and memory, suggesting their role in the neurogenic effects of antidepressants [16]. This evidence also indicated that TrkA/RAS/Raf/MERK, Insulin R or IGF-1R/PI3K/AKT/ERK and GR/PLC/PKC signaling pathways took important roles in neurite growth of PC12 cells.

In this study, we focused on the mechanism of action for neurite growth to elucidate the mechanism underlying *β*-cyc-induced neurite growth through the use of specific protein inhibitors and Western blotting. Furthermore, potential targets were predicted using specific inhibitor experiments, such as the Cellular Thermal Shift Assay (CETSA) and Drug Affinity Responsive Target Stability (DARTS). These findings implied that the dual-targeted impacts of *β*-cyc may involve IGF-1R and GR, ultimately leading to the activation of the PI3K/AKT and PLC/PKC signaling pathways.

## 2. Results

### 2.1. β-cyc Exhibits NGF-Mimic and NGF-Enhancing Effects on PC12 Cells

**Figure 1:**
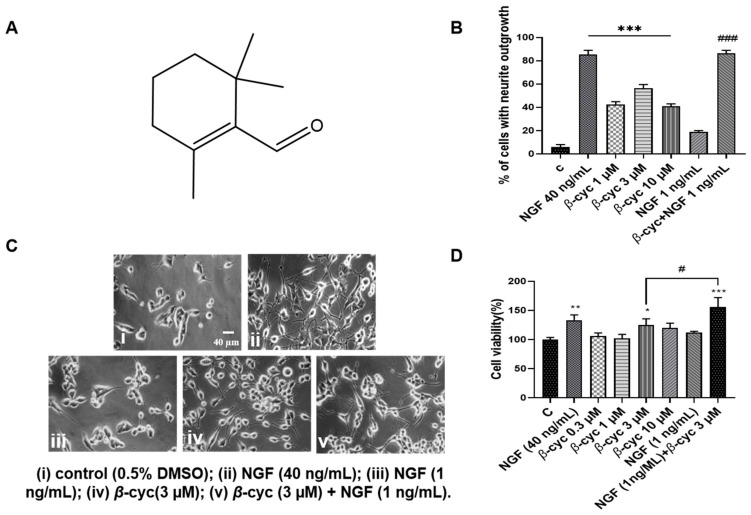
Effects of *β*-cyc on PC12 cells. (**A**) The structure of *β*-cyc. (**B**) Neurite outgrowth percentages were treated with varying doses of *β*-cyc alone or in combination with 1 ng/mL of NGF. (**C**) The morphological alterations after treatment for 48 h were observed by an inverted optical microscope. (**D**) Cell viability after exposure to different doses of *β*-cyc or *β*-cyc co-treated with 1 ng/mL of NGF. Each experiment was repeated three times. The data were expressed as a mean ± SEM. * *p* < 0.05, ** *p* < 0.01, and *** *p* < 0.001, compared with the negative control; ^#^ *p* < 0.05 and ^###^ *p* < 0.001, compared with the 3 µM *β*-cyc group.

Initially, we observed the neurotrophic activity of *β*-cyc in PC12 cells. The cells were treated with various concentrations of *β*-cyc (1, 3, or 10 µM) for 48 h, with DMSO serving as the negative control and NGF (40 ng/mL) acting as the positive control. The results showed that treatment with *β*-cyc significantly promoted neurite outgrowth. The percentages of PC12 cells with neurite outgrowth induced by *β*-cyc were 6.0% ± 1%, 42.3% ± 1.4%, 56.3% ± 1.6%, and 40.7% ± 1.15% at concentrations of 0, 1, 3, and 10 µM (*p* < 0.001, Figure 1B), respectively. Additionally, NGF-enhancing experiments demonstrated that treatment with 3 µM *β*-cyc significantly enhanced the percentage of PC12 cells with positive neurite outgrowth from 56.3% ± 1.6% to 86.3% ± 1.3% (*p* < 0.001, Figure 1B), when these cells were co-treated with low-dose NGF (1 ng/mL). The changes in cell morphology treated with 3 µM *β*-cyc alone or its combination with 1 ng/mL NGF were shown in Figure 1C. These results indicated that *β*-cyc exhibited both NGF-mimic and NGF-enhancing effects in PC12 cells.

To evaluate whether the neuritogenic activities were not associated with the toxicity of *β*-cyc, cell viability was evaluated in PC12 cells using the 3-(4,5-dimethylthiazol-2-yl)-2,5-diphenyltetrazolium bromide (MTT) bioassay. After treating the cells with *β*-cyc at doses of 0.03, 0.3, 3, and 10 µM, the cell viability was determined to be 106.04% ± 3.2%, 102.0% ± 3.4%, 125.3% ± 5.2%, and 120.3% ± 3.9%, respectively (Figure 1D). Additionally, when low-dose NGF was added to the group treated with 3 µM *β*-cyc, the cell viability increased significantly to 155.9% ± 8.1% (*p* < 0.001, Figure 1D). These results suggested that *β*-cyc alone did not exhibit cytotoxic effects, even at concentrations up to 10 µM. Moreover, the combination of 3 µM of *β*-cyc with 1 ng/mL of NGF at a low dose enhanced the viability of PC12 cells, similar to the effects of NGF at a dose of 40 ng/mL. Overall, the MTT bioassay results indicated that the tested concentrations of *β*-cyc did not demonstrate significant cytotoxicity.

### 2.2. Ras/Raf/MEK/ERK Signaling Pathway Involves in NGF-Mimic and NGF–Enhancing Effect of β-cyc

**Figure 2:**
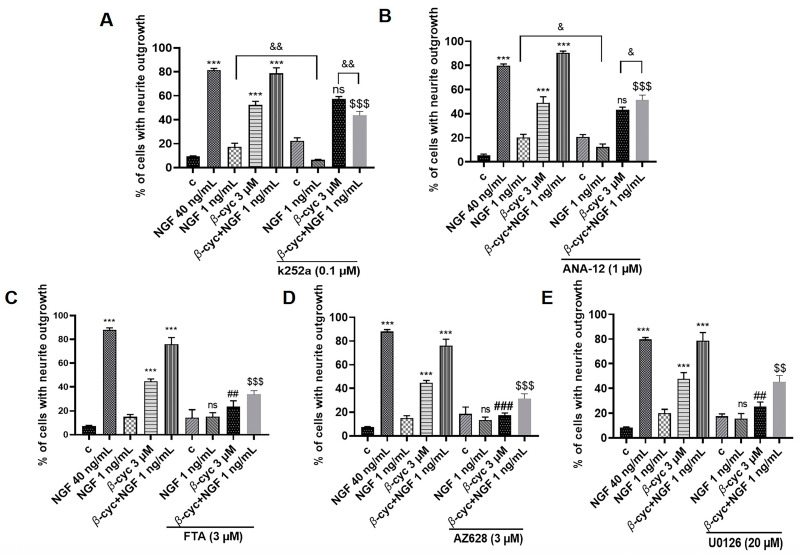
*β*-cyc modulation of the Ras/Raf signaling pathway in PC12 cells. (**A**–**E**) Effects of inhibitors of TrkA, TrkB, Ras, Raf, ERK (K252a, ANA-12, FTA, AZ628, U0126, respectively) on the neurite outgrowth induced by *β*-cyc, its combination with NGF, and NGF 1 ng/mL. Each experiment was repeated three times. The data were expressed as a mean ± SEM. *** *p* < 0.001, compared with the negative control; ^##^ *p* < 0.01 and ^###^ *p* < 0.001, compared with the 3 µM *β*-cyc group; ^$$^ *p* < 0.01 and ^$$$^ *p* < 0.001, compared with the combination group of *β*-cyc with low-dose NGF; ^&^ *p* < 0.05 and ^&&^ *p* < 0.01, ns means no significant difference.

Various neurotrophic factors, including NGF and BDNF, selectively interacted with the tyrosine kinase receptors TrkA and TrkB, activating multiple kinases to promote neuronal differentiation and survival [23,24]. Due to the NGF-mimic activity of *β*-cyc, it was hypothesized that *β*-cyc-induced neurite outgrowth may target the common receptor of NGF. Consequently, TrkA and TrkB inhibitors were initially used to block the effects of *β*-cyc. However, treatment with K252a and ANA-12, inhibitors of TrkA and TrkB, respectively, did not markedly alter the induction of neurite outgrowth induced by *β*-cyc alone. This suggested that the involvement of Trks in the neurogenic effect of *β*-cyc was limited in PC12 cells (Figure 2A,B). However, the NGF-enhancing activity of *β*-cyc was significantly inhibited by K252a and ANA-12, leading to notable differences between the *β*-cyc and *β*-cyc + NGF 1 ng/mL groups following treatment with K252a and ANA-12 (*p* < 0.01 and *p* < 0.05, respectively). These findings prompted us to speculate that K252a and ANA-12 effectively suppressed the NGF-enhancing activity of *β*-cyc by inhibiting low-dose NGF.

NGF initially stimulates TrkA and TrkB, which then induce differentiation in PC12 cells by arresting proliferation and promoting neurite outgrowth through the transient activation of Ras and Rap1-dependent ERK phosphorylation [25]. Specific inhibitors were further utilized to investigate the regulation of these signaling pathways. As depicted in Figure 2C–E, the addition of inhibitors of Ras, Raf, and ERK (FTA, AZ628 and U0126) significantly reduced the NGF-mimic effects induced by *β*-cyc, decreasing the percentage of neurite outgrowth from 51.5% ± 1.7% to 23.3% ± 2.5% (*p* < 0.01), 17.3% ± 1.0% (*p* < 0.001), and 25.3% ± 1.8% (*p* < 0.01), respectively. Similarly, these inhibitors decreased the enhancing effects of *β*-cyc combined with low-dose NGF, reducing the percentage from 78.7% ± 3.3% to 33.7% ± 1.6% (*p* < 0.001), 31.3% ± 2.1% (*p* < 0.001), and 45.3% ± 2.5% (*p* < 0.01), respectively. In conclusion, these findings suggested that the Ras/Raf signaling pathway may contribute to the neurogenic effect of *β*-cyc in PC12 cells, albeit independently of the Trk receptors.

### 2.3. IGF-1R/PI3K/AKT Signaling Pathway Takes an Important Role in NGF-Mimic Effect of β-cyc

Notably, NGF and BDNF also activate the PI3K/AKT/mTOR pathway [20]. Numerous studies have shown that phosphorylation of the IGF-1R receptor can initiate downstream changes in metabolic pathways, potentially leading to signal transduction cascades that regulate various cellular functions. These functions include promoting cell survival and neurite outgrowth via the PI3K/AKT signaling pathway [26]. Therefore, our objective was to investigate the potential involvement of this signaling pathway in the neuroprotective and neurite-promoting effects induced by *β*-cyc.

**Figure 3:**
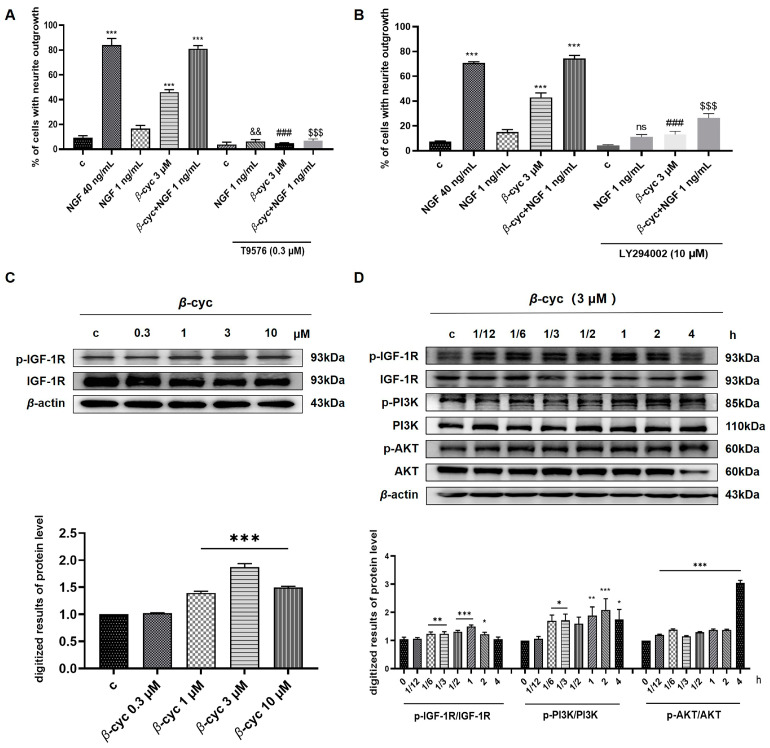
The IGF-1R signaling pathway plays a primary role in the neurogenesis effect of *β*-cyc. (**A**,**B**) Effects of the IGF-1R inhibitor T9576 and PI3K/AKT inhibitor LY294002 on the neurite outgrowth elicited by *β*-cyc, its combination with low-dose NGF, and NGF 1 ng/mL. (**C**) Western blot analysis and digitalization of IGF-1R and p-IGF-1R levels induced by *β*-cyc dose-dependently. (**D**) Western blot analysis and digitalized results of p-IGF-1R, IGF-1R, p-PI3K, PI3K, p-AKT, and AKT induced by *β*-cyc in a time-dependent experiment. Each experiment was repeated three times. The data were expressed as a mean ± SEM. * *p*< 0.05, ** *p*< 0.01, *** *p* < 0.001, compared with the negative control; ^###^ *p* < 0.001, compared with the 3 µM *β*-cyc group; ^$$$^ *p* < 0.001, compared with the combination group of *β*-cyc with low-dose NGF; ^&&^ *p* < 0.01, compared with the low-dose NGF, ns indicates no significant difference, compared with the low-dose NGF.

To explore the potential mechanisms of action of *β*-cyc, we employed the IGF-1R inhibitor T9576 to disrupt the activity induced by *β*-cyc. After treatment with T9576, the results demonstrated a substantial reduction in neurite outgrowth from 46.0% ± 1.0% to 4.6% ± 0.3% (*p* < 0.001) attributed to *β*-cyc (Figure 3A). Furthermore, treatment with LY294002, a PI3K/AKT inhibitor, notably reduced *β*-cyc-induced neurite growth from 44.3% ± 0.8% to 13.0% ± 1.3% (*p* < 0.001) (Figure 3B). Notably, both T9576 and LY294002 effectively inhibited neurite outgrowth induced by the combination of *β*-cyc and low-dose NGF in PC12 cells.

**Figure 4:**
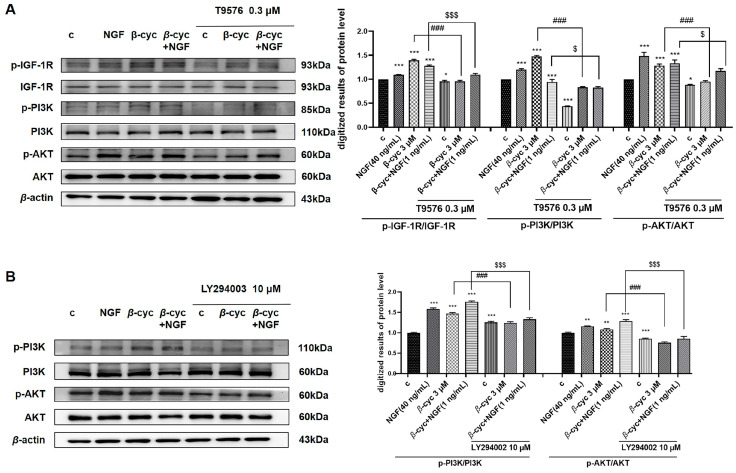
Related inhibitors on the modulation of the IGF-1R/PI3K/AKT pathway induced by *β*-cyc in PC12 cells. (**A**) The phosphorylation of the IGF-1R, PI3K, and AKT induced by *β*-cyc or its combination with low-dose NGF were downregulated by T9576. (**B**) The phosphorylation of PI3K and AKT stimulated by *β*-cyc or its combination with low-dose NGF was reduced by LY294002. Each experiment was repeated three times. The data were expressed as a mean ± SEM. * *p* < 0.05, ** *p* < 0.01 and *** *p* < 0.001, compared with the negative control; ^###^ *p* < 0.001, compared with the 3 µM *β*-cyc group; ^$^ *p* < 0.05 and ^$$$^ *p* < 0.001, compared with the combination group of *β*-cyc with low-dose NGF.

To further confirm the protein expression levels of members of this signaling pathway, we conducted a Western blot analysis. We examined the dose-dependent induction of IGF-1R phosphorylation by *β*-cyc and observed the optimal peak phosphorylation at a concentration of 3 μM (Figure 3C and Figure S2). Subsequently, we evaluated the time-dependent phosphorylation of IGF-1R, PI3K, and AKT after *β*-cyc treatment. The results revealed an increase in the levels of phosphorylated IGF-1R induced by *β*-cyc after 10 min, reaching a peak at 1 h. Similarly, phosphorylated PI3K levels escalated by *β*-cyc after 10 min, reaching a peak at 2 h. Additionally, the phosphorylation of AKT increased at 5 min and peaked at 4 h after *β*-cyc treatment. (Figure 3D and Figure S2). T9576 significantly reduced the levels of IGF-1R and the downstream proteins PI3K and AKT phosphorylation induced by *β*-cyc in the presence or absence of 1 ng/mL NGF (Figure 4A and Figure S3). Moreover, LY294002 diminished the phosphorylation of PI3K and AKT induced by *β*-cyc, both alone and in combination with low-dose NGF (Figure 4B and Figure S3). These findings indicated a close association between the IGF-1R/PI3K/AKT signaling pathway and the neurite outgrowth induced by *β*-cyc in PC12 cells.

### 2.4. GR/PLC/PKC Signaling Pathway Participates in the NGF-Mimic Effect of β-cyc

**Figure 5:**
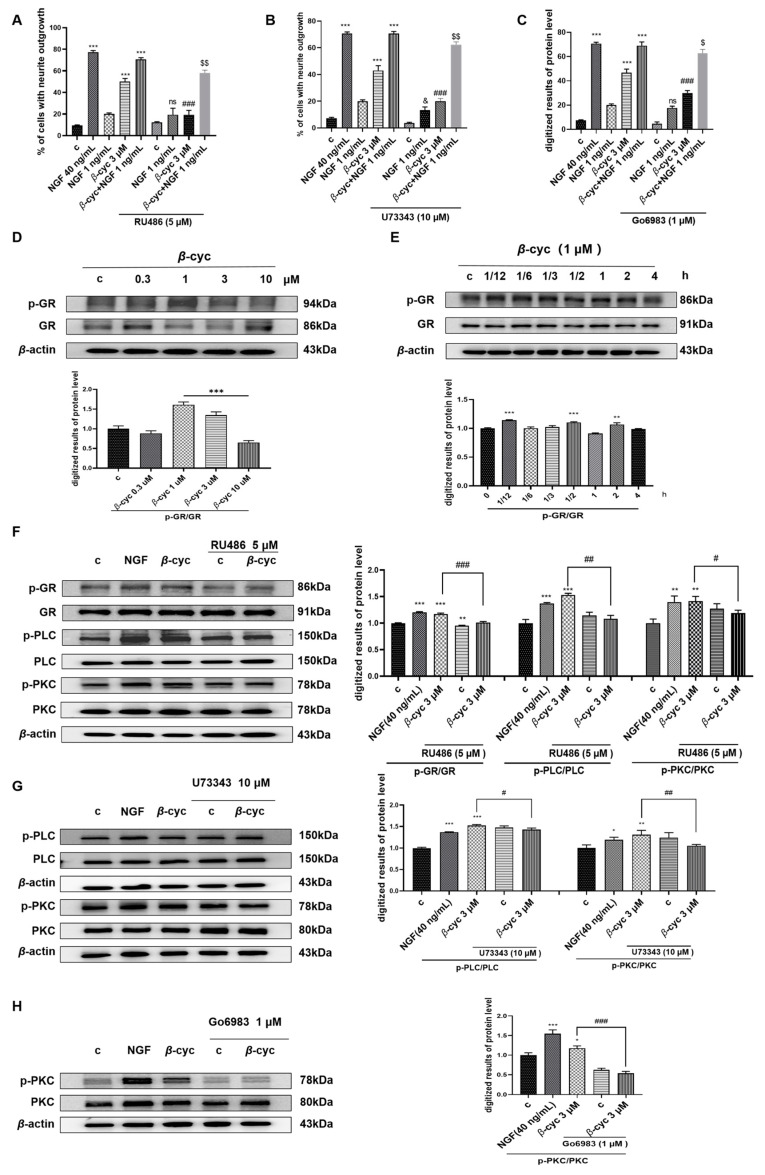
Modulation of the GR/PLC/PKC pathway by *β*-cyc in PC12 cells. (**A**–**C**) Effects of inhibitors of GR, PLC, and PKC (RU486, U73343, and Go6983) on the neurite outgrowth induced by *β*-cyc, its combination with low-dose NGF, and NGF 1 ng/mL. (**D**,**E**) Western blotting and digitalized result of p-GR, GR in dose-dependent (**D**) and time-dependent (**E**) experiments induced by *β*-cyc. (**F**) The induction of phosphorylation in GR, PLC, and PKC by *β*-cyc and RU486. (**G**) The induction of phosphorylation in PLC and PKC by *β*-cyc and U73343. (**H**) Phosphorylation of PKC by *β*-cyc and Go6983. Each experiment was repeated three times. The data were expressed as a mean ± SEM. * *p* < 0.05, ** *p* < 0.01 and *** *p* < 0.001, compared with the negative control; ^#^ *p* < 0.05, ^##^ *p* < 0.01 and ^###^ *p* < 0.001, compared with the 3 µM *β*-cyc group; ^$^ *p* < 0.05 and ^$$^ *p* < 0.01, compared with the combination group of *β*-cyc with low-dose NGF; ^&^ *p* < 0.05, compared with the low-dose NGF, ns indicates no significant difference, compared with the low-dose NGF.

The participation of GR in the neurogenic activity of PC12 cells and its regulation of a suite of genes crucial for neuronal structural control has been demonstrated [27]. Therefore, the potential mechanism of action of *β*-cyc was further elucidated by using the GR inhibitor RU486. Following pretreatment with RU486, the cell proportion with neurite outgrowth decreased significantly after 48 h of drug exposure, with a decrease from 50.0% ± 1.5% to 19.3% ± 2.0% (*p* < 0.001) (Figure 5A).

Considering the pivotal role of PLC/PKC pathway, which is downstream of GR protein, plays in cell survival and differentiation as documented in reference [28]. This investigation employed the inhibitors of PLC and PKC (U73343 and Go6983) to explore the neurite-promoting effects of *β*-cyc. The results demonstrated that neurite outgrowth induced by *β*-cyc significantly decreased following the addition of U73343 or Go6983, resulting in percentages of 20.0% ± 1.0% and 30.0% ± 1.0% (*p* < 0.001), respectively, compared to the initial 50.0% ± 1.5% (Figure 5B,C). This finding suggested a close association between the activation of the PLC/PKC pathway and the neurogenic response of PC12 cells upon stimulation by *β*-cyc.

The phosphorylation levels of GR, PLC, and PKC were measured and analyzed at the protein level via Western blotting. The results indicated that the highest ratio of phosphorylated GR to total GR protein was achieved following treatment with 1 µM *β*-cyc (Figure 5D and Figure S4). Subsequently, the time-dependent phosphorylation of GR induced by *β*-cyc at 1 µM was investigated, revealing that GR phosphorylation peaked at 30 min (Figure 5E and Figure S4). Following pretreatment with RU486 and subsequent *β*-cyc treatment, a significant reduction in the phosphorylation of GR and its downstream proteins PLC and PKC was observed (Figure 5F and Figure S4). Moreover, the phosphorylation of PLC and PKC, triggered by *β*-cyc, was attenuated by U73343. Concurrently, Go6983 led to a reduction in PKC phosphorylation induced by *β*-cyc (Figure 5G,H and Figure S4). Overall, these results robustly implied the participation of the GR/PLC/PKC pathway in the facilitation of neurite outgrowth within PC12 cells mediated by *β*-cyc.

### 2.5. β-cyc Increases the Thermal Stable Ability of IGF-1R in CETSA

Previous inhibitor experiments revealed that ANA-12 did not significantly inhibit *β*-cyc, suggesting that TrkB is not a priority for targeting *β*-cyc activity. In contrast, T9576 and RU486, particularly T9576, exhibited noticeable inhibitory effects on the neurogenic action of *β*-cyc. This led us to hypothesize that the IGF-1R or GR proteins may serve as potential targets for *β*-cyc.

**Figure 6:**
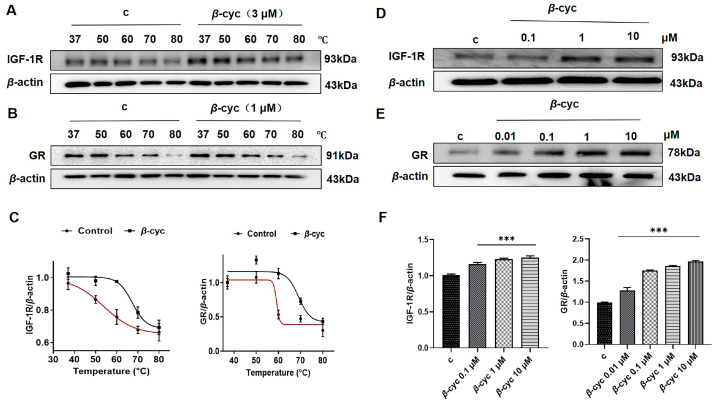
Target prediction of *β*-cyc by CETSA. (**A**,**B**) The interaction between *β*-cyc and IGF-1R and GR protein was studied using CETSA. *β*-cyc at 3 and 1 μM were assessed to investigate the expression changes in IGF-1R and GR protein at varying temperatures by Western blotting. (**C**) Quantification of the data for panels (**A**,**B**). (**D**,**E**) The modified CETSA method was utilized to explore the interaction between *β*-cyc and the IGF-1R protein/GR protein following alterations in the concentration of *β*-cyc. (**F**) Quantitative analysis for panels D and E. Each experiment was repeated three times. The data were expressed as a mean ± SEM. *** *p* < 0.001, compared with the negative control.

Consequently, the binding between IGF-1R and *β*-cyc was further confirmed to the potential targets of *β*-cyc using a CETSA. Briefly, cells were treated with either DMSO or *β*-cyc, heated from 37 °C to 80 °C, and then analyzed through immunoblotting using a specific antibody against IGF-1R. The results showed that the IGF-1R protein exhibited a significant shift in thermal stability following *β*-cyc treatment, which was characterized by a substantial increase in thermal stability. However, in the control group, protein degradation occurred as the temperature increased (Figure 6A,C and Figure S5).

Consistent with our hypothesis, GR proteins also experienced a shift in thermal stability after *β*-cyc treatment according to the CETSA (Figure 6B,C and Figure S5). These findings partially validate that IGF-1R and GR may indeed be potential dual target proteins of *β*-cyc. Based on these findings, a modified CETSA approach was employed, involving keeping the temperature fixed while varying the concentrations of the drug to assess the stability of the drug-protein interactions. For the IGF-1R protein, the concentrations of *β*-cyc-treated cells ranged from 0.1 μM to 10 μM at the optimal temperature of 60 °C. Western blot analysis indicated an increase in the stability of the interaction between *β*-cyc and the IGF-1R protein with increasing concentrations of *β*-cyc, demonstrating a clear dose dependency (Figure 6D,F and Figure S5). Similarly, the stability of the GR proteins increased with various concentrations of *β*-cyc from 0.01 μM to 10 μM, indicating a marked dose dependency (Figure 6E,F and Figure S5). These findings collectively further confirmed the dual-target effects of IGF-1R and GR as potential target proteins of *β*-cyc.

### 2.6. β-cyc Enhances the Stability of IGF-1R and GR Proteins for Pronase E During DARTS Analysis

**Figure 7:**
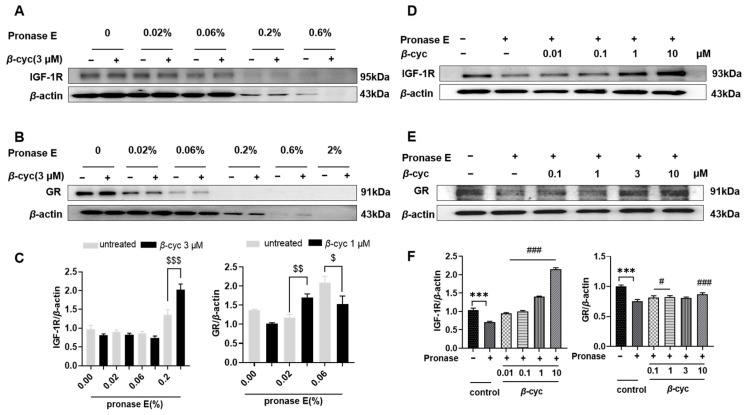
Target prediction of *β*-cyc by DARTS assay. (**A**) Western blot analysis of IGF-1R and (**B**) GR protein level after adding different concentrations of pronase E. (**C**) Quantification of the data obtained from panels (**A**,**B**). (**D**) Protein level of IGF-1R after adding different concentrations of *β*-cyc (0.1, 1, 3, 10 µM) by Western blotting. IGF-1R was normalized with *β*-actin. (**E**) Western blot analysis of GR protein level after adding different concentrations of *β*-cyc (0.01, 0.1, 1, 10 µM). GR was normalized with *β*-actin. (**F**) Quantification of the data obtained from panels D-E. Each experiment was conducted three times. ^$^ *p* < 0.05, ^$$^ *p* < 0.01 and ^$$$^ *p* < 0.001 in comparison to the untreated group; *** *p* < 0.001 compared to the negative control without pronase E; ^#^ *p* < 0.05 and ^###^ *p* < 0.001 compared with the negative control with pronase E.

Based on previous findings, initial validation focused on the IGF-1R protein using DARTS assays. The protein levels of IGF-1R in the group exposed to 3 μM *β*-cyc for 3 h were significantly greater than those in the untreated group when the concentration of pronase E was 0.2% (*p* < 0.001) (Figure 7A,C and Figure S6). Importantly, the levels of IGF-1R in the group exposed to various concentrations of *β*-cyc continued to increase and remained higher than those in the untreated group under identical conditions (*p* < 0.001) (Figure 7C,D and Figure S6). These results indicated that the effect was dose-dependent as the drug concentration increased.

Furthermore, compared with those of GR, the protein stability of GR increased in a concentration-dependent manner following *β*-cyc treatment, although the dose dependency and increase in IGF-1R levels were more pronounced (Figure 7B,E,F and Figure S6). Ultimately, these findings provided further support for the notion that IGF-1R and GR are potential target proteins of *β*-cyc.

## 3. Discussion

Recent failures in clinical trials investigating amyloid-beta clearance have generated interest in alternative therapeutic strategies for AD. Research suggests that A*β* plaques may have a protective role in the early stages of AD, although their correlation with disease severity appears to be weak. Treatments focused on A*β* plaques, such as the γ-secretase inhibitor semagacestat and the *β*-secretase inhibitor verubecestat, have shown inconclusive efficacy [8]. However, further investigations are needed to determine the effectiveness of other AD drugs, such as aducanumab and GV-971. Therefore, the development of clinical drugs for AD requires the exploration of diverse mechanisms and targets. In particular, neurogenesis has become the new strategy for the treatment of AD.

Lavender essential oil, demonstrating neuroprotective effects and promising cognitive improvement, has the anti-AD potential supported by both dementia model rats and clinical studies [29,30,31,32]. However, the material basis for producing anti-AD effects is still unclear, and the mechanism of activity still needs to be elucidated. Therefore, we employed a PC12 cell line, a well-established model for studying neuronal differentiation, to perform bioactivity-guided isolation and purification of lavender essential oil, leading to the identification of a small molecule, *β*-cyc. The morphological alterations observed in PC12 cells after treatment with *β*-cyc or *β*-cyc + low dose of NGF in Figure 1 suggested that *β*-cyc not only has NGF-mimic effects but also has NGF-enhancing effects. These results are consistent with our previous findings [13,14,15]

To clarify the mechanism of action of *β*-cyc, we first focused on the signaling pathways related to the neurogenic effects to do an investigation with specific protein inhibitors and western blotting analysis. Given that TrkA and TrkB serve as specific transmembrane targets for the neurotrophins NGF and BDNF, respectively, we initiated a screening process utilizing inhibitors targeting TrkA, TrkB, Ras, Raf, ERK, SAPK/JNK, and p38 MAPK. Additionally, inhibitors directed against *β*-arrestin, PKA, and GSK-3*β* were also included owing to their close association with the promotion of neurite outgrowth. The changes in NGF mimics or enhancer effects of *β*-cyc on PC12 cells after giving these inhibitors in Figure 1 and Figure S1 indicated that TrkA and TrKB were not involved in the NGF-mimic effects of *β*-cyc, whereas Ras, Raf, and ERK may be implicated in the neurogenic effect of *β*-cyc.

IGF-1 plays an essential role in CNS development and maturation. Recent preclinical and clinical evidence indicate that IGF-1 not only regulated growth and development but also prevented neuronal death mediated by amyloidogenesis, neuroinflammation, and apoptosis through modulation of PI3/Akt kinase, mTOR, and MAPK/ERK signaling [33]. Meanwhile, some evidence indicated that GR regulates a series of important genes for neuronal structure and plasticity and is involved in the neuritogenic activity in PC12 cells [13,28]. Since the PI3K/AKT and PLC/PKC signaling pathways demonstrate significant involvement in neurogenesis [34,35]. Therefore, we checked whether these two signaling pathways were involved in the NGF-mimic effects of *β*-cyc with specific protein inhibitors and western blotting analysis. As we expected, the significant suppression of the neurogenic effect of *β*-cyc by IGF-1R and GR inhibitors, as well as additional inhibitors targeting their downstream proteins PI3K/AKT, PLC, and PKC in Figure 3, Figure 4 and Figure 5, elucidated that IGF-1R/PI3K/AKT and GR/PLC/PKC signaling pathways took important roles in the neurogenic effects of *β*-cyc in PC12 cells. Interestingly, the mechanism of the action of *β*-cyc for its NGF-mimic effect was different from that of compounds such as AMA, Lindersin B, and CuB. AMA, a secoiridoid glycoside from *Gentiana rigescens*, promotes neurogenesis by activating the INSR/PI3K/AKT and Ras/Raf/MEK signaling pathways in PC12 cells [14]. Lindersin B, a cucurbitane triterpenoid from *Lindernia crustacea*, stimulated neuritogenesis via the activation of the TrkA/PI3K/ERK signaling pathway [15]. CuB, a terpene from *Cucumis melo* (commonly known as Tiangua Di in Chinese), induced neurite growth by targeting cofilin and altering the GR and TrkA pathways [13].

Identification of the target proteins of small molecules is crucial to explore their mechanism of action. Therefore, we screened potential target proteins of *β*-cyc with specific protein inhibitor experiments and label-free protein target techniques, such as DARTS and CETSA. The effects of IGF-1R and GR inhibitors on the NGF-mimic effect of *β*-cyc in Figure 4 and Figure 5 and the increase of thermal stability and pronase E stability of IGF-1R and GR of CETSA and DARTS in Figure 6 and Figure 7 demonstrated that IGF-1R and GR might be potential target protein of *β*-cyc to produce NGF-mimic effect on PC12 cells. Regrettably, we failed to get direct evidence of which *β*-cyc bound with IGF-1R or GR by SPR analysis.

Emerging research suggests that combination therapy utilizing molecules with diverse structures and mechanisms holds great promise as an approach to treating AD. In contrast, the use of multiple single-target drugs often leads to increased medication burden and a greater risk of cumulative toxicity and side effects. Dual-targeted therapies are considered potential solutions for overcoming drug resistance, as they offer synergistic benefits with reduced adverse effects and decreased toxicity. Progress in dual-target drug development has been evident in clinical trials. For instance, Blinatumomab, which targets CD19 and CD3, obtained FDA approval in 2014 for the treatment of acute B lymphoblastic leukemia [36]. Other notable developments include the PD-L1/CTLA-4 bispecific antibody KN046, which is effective in the treatment of solid tumors, and AK104, targeting PD-1 and CTLA-4, demonstrated increased efficacy with fewer adverse events [37,38]. This movement toward dual-target and multitarget drugs represents a crucial research direction. These results provided insights into the combination that the use of these molecules may have in increasing the therapy effect for AD.

## 4. Materials and Methods

### 4.1. Antibodies and Reagents

DMSO (CAT No.: D8418), NGF (CAT No.: N2513), TrkA inhibitor (k252a, CAT No.: 420298-M), MEK/ERK inhibitor (U0126, CAT No.: 19-147), PI3K inhibitor (LY294002, CAT No.: 440202), PKC inhibitor (Go6983, CAT No.: 365251), and GR inhibitor (RU486, CAS No.: 84371-65-3) were bought from Sigma-Aldrich Co, Boston, MA, USA. The INSR inhibitor (CAT No.: sc-221730), IGF-1R inhibitor (T9576, CAS No.: 477-47-4), Raf inhibitor (AZ628, CAT No.: sc-364418), and PLC inhibitor (U-73343, CAT No.: sc-201422) were obtained from Santa Cruz Biotechnology, Dallas, TX, USA. The Ras inhibitor (FTA, Item No.: 17474) was obtained from Cayman Chemical, Ann Arbor, MI, USA. The pronase E (CAT No.: HY-114158) was obtained from MedChemExpress, Shanghai, China, while the TrkB inhibitor (ANA-12, CAT No.: S7745) was obtained from Selleck, Shanghai, China.

The antibodies against IGF-1R (CAT No.: 3027S), phospho-IGF-1R (Tyr1131, CAT No.: 3024S), PI3 kinase (CAT No.: 4249S), phospho-PI3 kinase (Tyr458, CAT No.: 4228S), AKT (CAT No.: 9272S), phospho-AKT (Ser473, CAT No.: 9271S), GR (CAT No.: 12041T), PLC (CAT No.: 14008S), phospho-PKC (CAT No.: 2261S), and PKC (CAT No.: 38168S) were obtained from Cell Signaling Technology, Boston, MA, USA, and the antibodies against phospho-GR (CAT No.: AF2004) and phospho-PLC γ (CAT No.: AF4454) were obtained from Affinity Biosciences, Cincinnati, OH, USA. The β-actin antibody (CAT No.: CW0096M), secondary antibodies horseradish peroxidase-linked anti-mouse (CAT No.: CW0102S) and anti-rabbit IgGs (CAT No.: CW0103S), and the pico-ECL Western blot chemiluminescence detection kit (CAT No.: CW0049M) were obtained from Beijing CoWin Biotech Company, Beijing, China, respectively. In addition, it is with great regret that we cannot replicate the study in hippocampus-specific cell lines in this revision because there is no cell line in our laboratory now. We accepted your suggestion and use this cell line as the model in our study in the future.

### 4.2. Extraction and Isolation

Lavender oil was obtained from Thursday Plantation, Rydalmere, NSW, Australia. Chromatographic separation was performed on 1.5 g of lavender oil using a silica gel column, eluting with a gradient of *n*-hexane/CH_2_Cl_2_ (10:0, 7:3, 5:5, 3:7, 0:10), resulting in six fractions. The most active fraction (206.1 mg) was washed out with CH_2_Cl_2_/MeOH (7:3) and further purified by HPLC using a Cosmosil 5C18-MS-II packed column (10 × 250 mm) (Nacalai Tesque, Tokyo, Japan) with a linear gradient of MeOH/H_2_O: 40:60–100:0 over 80 min with a detection wavelength set to 210 nm and using a flow rate set at 3 mL/min. This process yielded an active compound (13.6 mg, retention time = 49.4 min), which was identified as *β*-cyc by comparing its ^1^H NMR data with the previous literature [39]. ^1^H NMR (500 MHz, CDCl_3_) data: *δ* 10.11 (s, 1H, CO-H), 2.17 (t, *J* = 6.3 Hz, 2H, CH_2_), 2.08 (s, 3H, CH_2_), 1.63–1.58 (m, 2H, CH_2_), 1.44–1.41 (m, 2H, CH_2_), 1.18 (s, 6H, 2 × CH_2_). The structure of *β*-cyc is shown in Figure 1A.

### 4.3. Evaluation of the Neuritogenic Activity of PC12 Cells

The evaluation of neuritogenic activity in the PC12 cells was described in our previous publication [15]. Around 5 × 10^4^ cells/well were cultured in a 24-well microplate under suitable conditions. After that, the medium in each well was exchanged with 1 mL of DMEM containing the test compound dissolved in DMSO or 0.5% DMSO alone. NGF at a concentration of 40 ng/mL served as the positive control. After 48 h, 100 cells were randomly selected and counted in triplicate. For inhibitor screening, cells were initially pretreated with 500 µL of DMEM that contained a specific inhibitor for 30 min. After that, an additional 500 µL of DMEM with either the test compound or 0.5% DMSO was introduced. The alterations in cellular morphology were assessed in each well after 48 h, and the percentage of positive cells in the selected area was quantified.

### 4.4. Cell Viability by MTT Bioassay

The cells were incubated with different concentrations of *β*-cyc for 48 h or with a combination of *β*-cyc (3 µM) and low-dose NGF (1 ng/mL). After removing the original culture medium, 500 µL of medium containing 200 µg/mL MTT was added to each well and incubated at 37 °C for 2 h. Subsequently, the medium per well was substituted with 200 µL of DMSO to dissolve the formazan crystals, which were then detected at 570 nm using a spectrophotometer.

### 4.5. Western Blot Analysis

Western blot analysis was conducted in accordance with the methods outlined in previous studies [13]. Briefly, the cells were homogenized in a lysis buffer containing 1% protease and phosphatase inhibitors to extract proteins. Protein concentrations were measured using the BCA assay, and all samples were denatured at 100 °C for 10 min. Subsequently, 20 µg of protein from each sample was uploaded on a sodium dodecyl sulfate–polyacrylamide gel (SDS–PAGE). The gel electrophoresis was executed at 80 V for 15 min, followed by 120 V for 60 min. The proteins were transferred onto a polyvinylidene difluoride (PVDF) membrane and then blocked in 5% skim milk for 60 min. The PVDF membrane underwent overnight incubation at 4 °C with primary antibodies, while the anti-*β*-actin antibody was employed as the normalization control. Following washing, the membrane underwent incubation with a secondary antibody for 45 min. Finally, the membrane was washed and exposed using a chemiluminescence detection kit (Beijing Cowin Biotech Company, Beijing, China). The protein bands were quantified using ImageJ software (Version 1.42q, National Institutes of Health, Rockville, MD, USA).

### 4.6. CETSA

CETSA is a widely utilized method for target validation that exploits changes in protein thermal stability following interaction with ligands to identify target proteins. Initially, 2 × 10^6^ cells were cultured in 60 mm dishes and incubated for 24 h. Subsequently, a control group was treated with 0.5% DMSO, while additional dishes were treated with 3 µM of *β*-cyc. After 24 h of incubation, the proteins were extracted and subjected to heating using a Veriti 96-well thermal cycler at varying temperatures. Subsequently, changes in the protein expression of IGF-1R and GR were assessed through Western blotting.

### 4.7. DARTS

DARTS is a technique utilized to identify interactions between drug molecules and proteins. Specifically, lysates derived from PC12 cells were incubated with or without *β*-cyc (3 µM) or *β*-cyc (1 µM) for 4 h at room temperature. Subsequently, the lysates were divided into several portions and treated with varying concentrations of pronase E for 25 min at room temperature. To terminate the digestion process, the samples were promptly boiled at 100 °C for 10 min following the addition of loading buffer. Finally, Western blot analysis was conducted to identify any alterations in the protein expression of IGF-1R or GR. Upon determining the optimal concentration of pronase E, different concentrations of *β*-cyc, lysates, and pronase E were co-incubated, following the incubation steps as described above. Finally, protein expression was analyzed using Western blotting.

### 4.8. Quantification and Statistical Analysis

Data from three independent experiments, each performed in triplicate and expressed as mean ± SEM, were subjected to one-way ANOVA and subsequent Tukey’s post hoc test via GraphPad Prism 8.0.2 software (GraphPad Software, San Diego, CA, USA). *p* < 0.05 was considered statistically significant.

## 5. Conclusions

**Figure 8:**
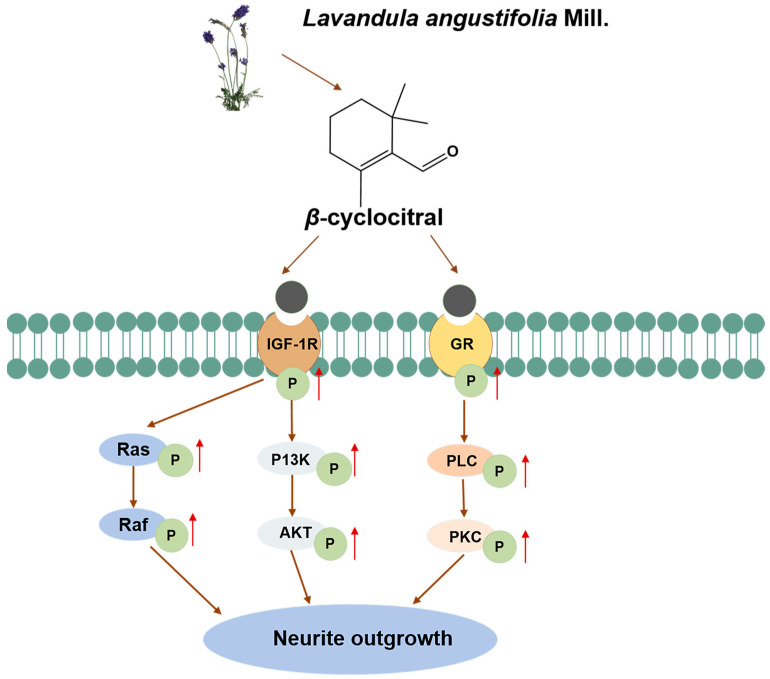
Proposed mechanism of action for *β*-cyc. *β*-cyc promotes neurogenesis by targeting IGF-1R and GR to activate downstream PI3K/AKT and PLC/PKC signaling pathways. The up arrow represents an increase in protein phosphorylation level.

In conclusion, *β*-cyc from Lavender has novel NGF-mimic and enhancer effects on PC12 cells. This compound existed the NGF-mimic and enhancer effect on PC12 cells via targeting of the IGF-1R and GR protein and regulation of the PI3K/AKT/Ras/Raf/ERK and GR/PLC/PKC signaling pathways (Figure 8). This study indicated the potential applications of *β*-cyc for its neurogenesis effect and provided evidence for the treatment of neurodegenerative diseases. Furthermore, the leading compound will be determined via modification of *β*-cyc, and the study of the chemical structure -bioactivity relationship and the anti-AD effects and the mechanism of actions of the leading compound will be evaluated and clarified with the AD animal model.

## Data Availability

The data supporting the conclusions of this study can be acquired from the corresponding author upon a reasonable request.

## Supplementary Materials

The following supporting information can be downloaded at: https://www.mdpi.com/article/10.3390/ijms25189763/s1.

## Abbreviations

Alzheimer’s disease, AD; amarogentin, AMA; Analysis of variance, ANOVA; beta amyloid, Aβ bicinchoninic acid, BCA; brain-derived neurotrophic factor, BDNF; cellular thermal shift assay CETSA; Cucurbitacin B, CuB; beta-Cyclocitral, β-cyc; drug affinity responsive target stability, DARTS; dulbecco’s modified eagle medium, DMEM; dimethyl sulfoxide, DMSO; extracellular signal-regulated kinase, ERK; glucocorticoid receptor, GR; insulin-like growth factor-1 receptor, IGF-1R; 3-(4,5-Dimethylthiazol-2-yl)-2,5-diphenyltetrazolium bromide, MTT; N-Methyl-D-Aspartate, Nerve growth factor, NGF; NMDA; phosphatidylinositol 3 kinase, PI3K; polyvinylidene difluoride, PVDF; phospholipase C, PLC; rat sarcoma, Ras; raf proto oncogene serine threonine protein kinase, Raf; Standard error of the mean, SEM; serine/thronin-protein kinase, AKT; sodium dodecyl sulfate–polyacrylamide gel, SDS–PAGE; tyrosine kinase, Trk; tyrosine kinase A, TrkA; tyrosine kinase B, TrkB.
