# Supplementary material for: IGF-1 and Glucocorticoid Receptors Are Potential Target Proteins for the NGF-Mimic Effect of β-Cyclocitral from Lavandula angustifolia Mill. in PC12 Cells
Source: Int J Mol Sci. 2024 Sep 10;25(18):9763. doi: 10.3390/ijms25189763 (PMC11432015; doi:10.3390/ijms25189763)
Supplement: Supplementary file 1 [file ijms-25-09763-s001.zip › ijms-3156189-SI.pdf]

Supplementary Information

IGF-1 and Glucocorticoid Receptors Are Potential Target Proteins for the NGF-mimic effect of β-Cyclocitral from *Lavandula angustifolia Mill* in PC12 Cells

Chenyue An, Lijuan Gao, *, Lan Xiang, * and Jianhua Qi, *

College of Pharmaceutical Sciences, Zhejiang University, 866 Yu Hang Tang Road, Hangzhou, China

*Correspondence should be addressed to Lijuan Gao, K923146@zju.edu.cn; Lan Xiang, lxiang@zju.edu.cn; Jianhua Qi; qijianhua@zju.edu.cn

The preliminary screening for inhibitor trails

Materials:

β-arrestin inhibitor (ML192), PKA inhibitor (H-89), SAPK/JNK inhibitor (SP600125), and p38 MAPK inhibitor (SB203580) were bought from MedChemExpress; GSK-3*β* inhibitor (LiCl) were bought from Sigma–Aldrich Co.

Results:

To confirm the relationship between *β*-cyc and the signalling pathways related to GPCR and MAPK, we used inhibitors of *β*-arrestin, PKA, GSK-3*β*, SAPK/JNK, and p38 MAPK (ML192, H-89, LiCl, SP600125, SB203580, respectively) to study the role of *β*-cyc in promoting neurite outgrowth. The results revealed that, except for a slight inhibitory effect of the p38 MAPK inhibitor SB203580 on the NGF-enhancing activity of *β*-cyc ($p < 0.05$), whereas the involvement of other proteins appeared to be less significant or unrelated.

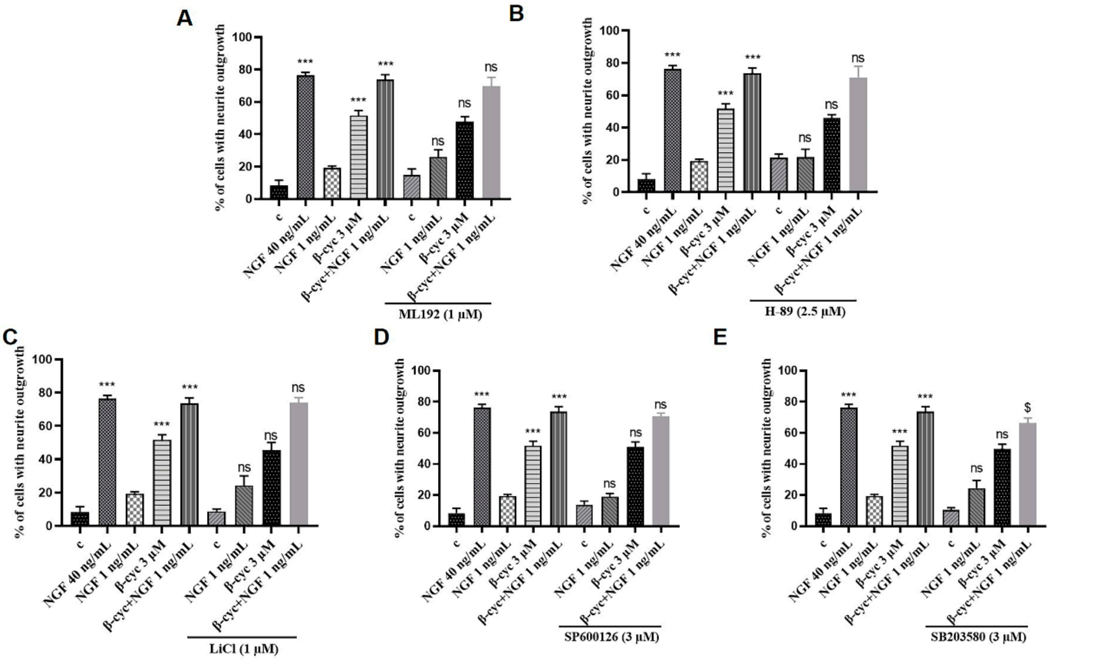

Figure S1. Inhibitors screening test of *β*-cyc-induced neurite outgrowth on MAPK and GPCR signaling pathway in PC12 cells. (A-E) Effects of inhibitors of *β*-arrestin, PKA, GSK-3*β*, SAPK/JNK, p38 MAPK, (ML192, H-89, LiCl, SP600125, SB203580, respectively) on the neurite outgrowth induced by *β*-cyc and its combination with NGF. Each experiment was repeated three times. The data were expressed as a mean ± SEM. $^{***}p < 0.001$, compared with the negative control; $^{\$}p < 0.05$, compared with the combination group of *β*-cyc with low-dose NGF.

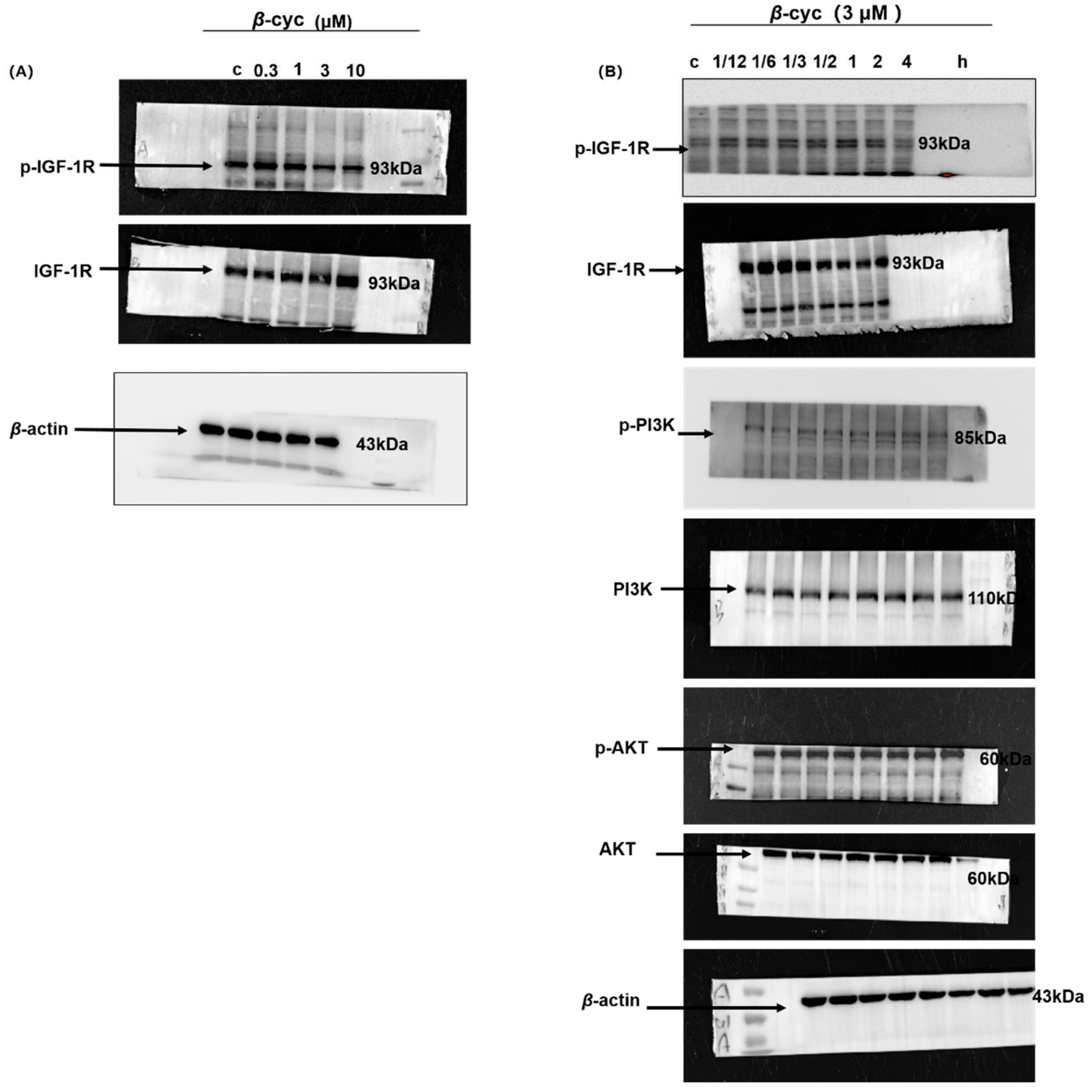

Figure S2: (A,B) Original data of western blot analysis of p-IGF-1R, IGF1R, p-PI3K, PI3K, p-AKT, AKT and β-actin in Figure 3C and 3D.

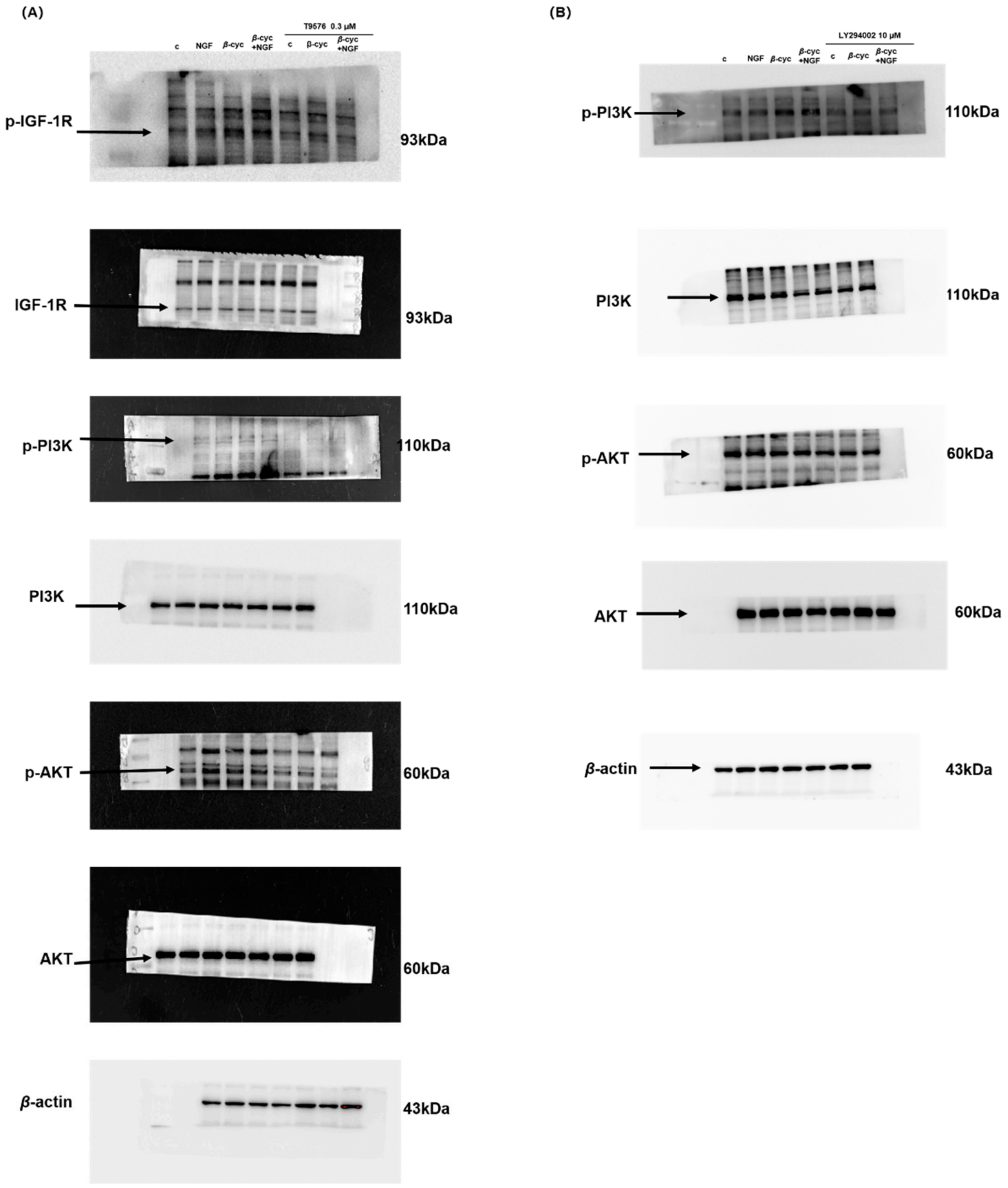

Figure S3: (A,B) Original data of western blot analysis of p-IGF-1R, IGF1R, p-PI3K, PI3K, p-AKT, AKT and *β*-actin in Figure 4A and 4B.

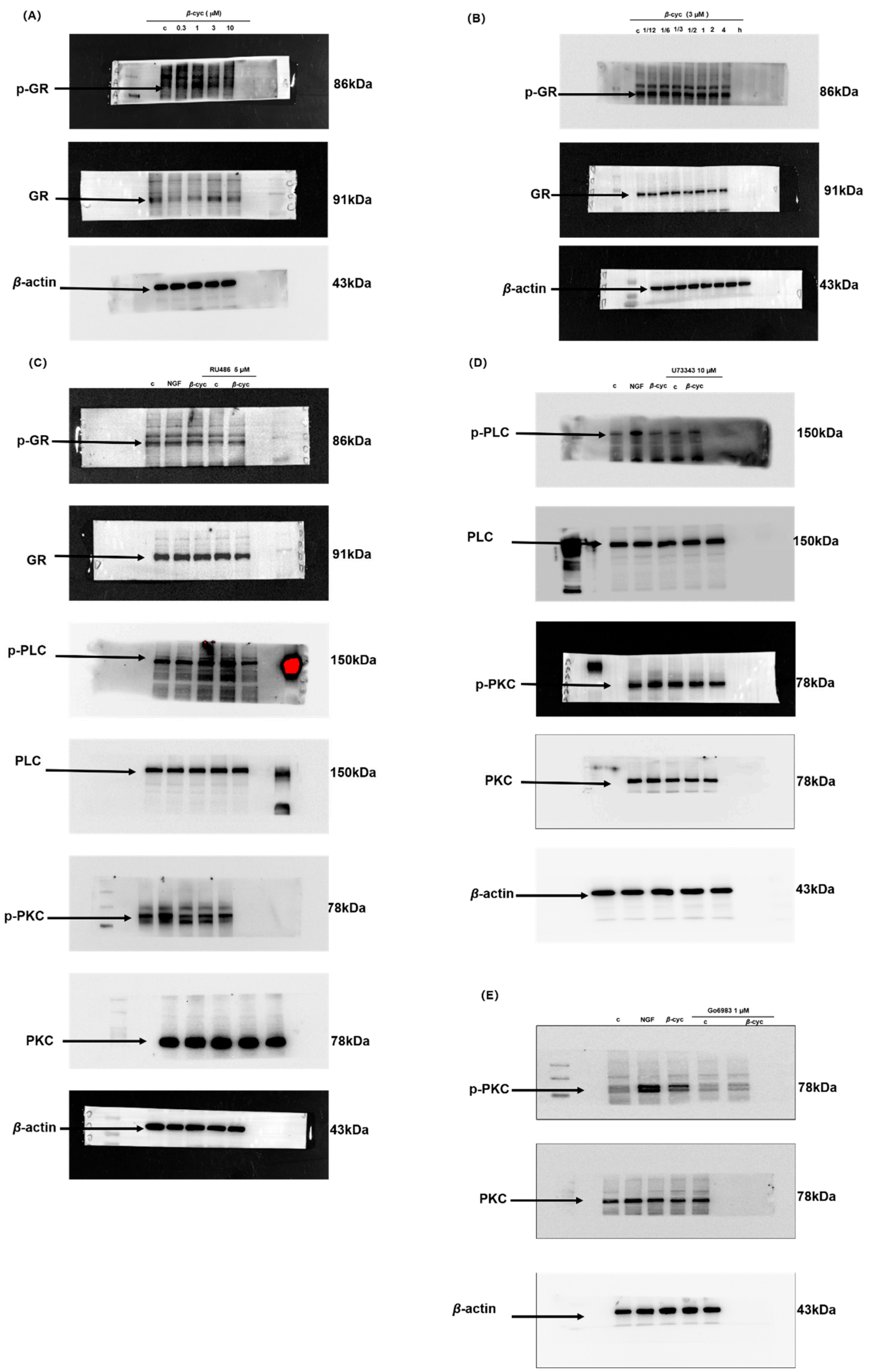

Figure S4: (A-D) Original data of western blot analysis of p-GR, GR, p-PLC, PLC, p-PKC, PKC and β-actin in Figure 5D-5H.

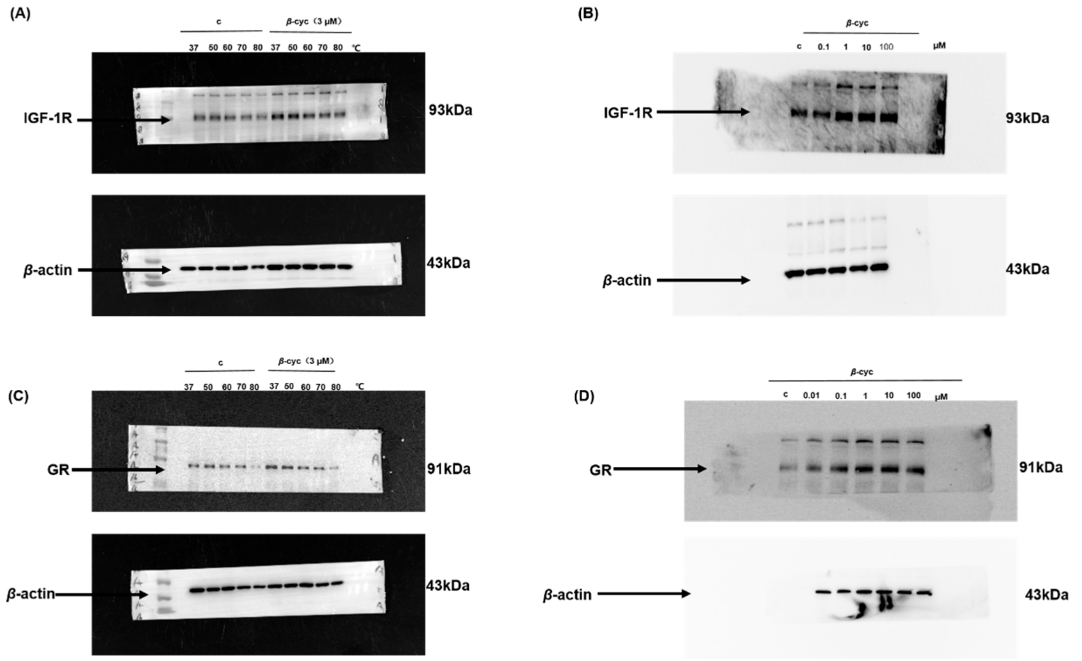

Figure S5: (A,B) Original data of western blot analysis of IGF-1R, GR and *β*-actin in Figure 6A-B and 6D-E.

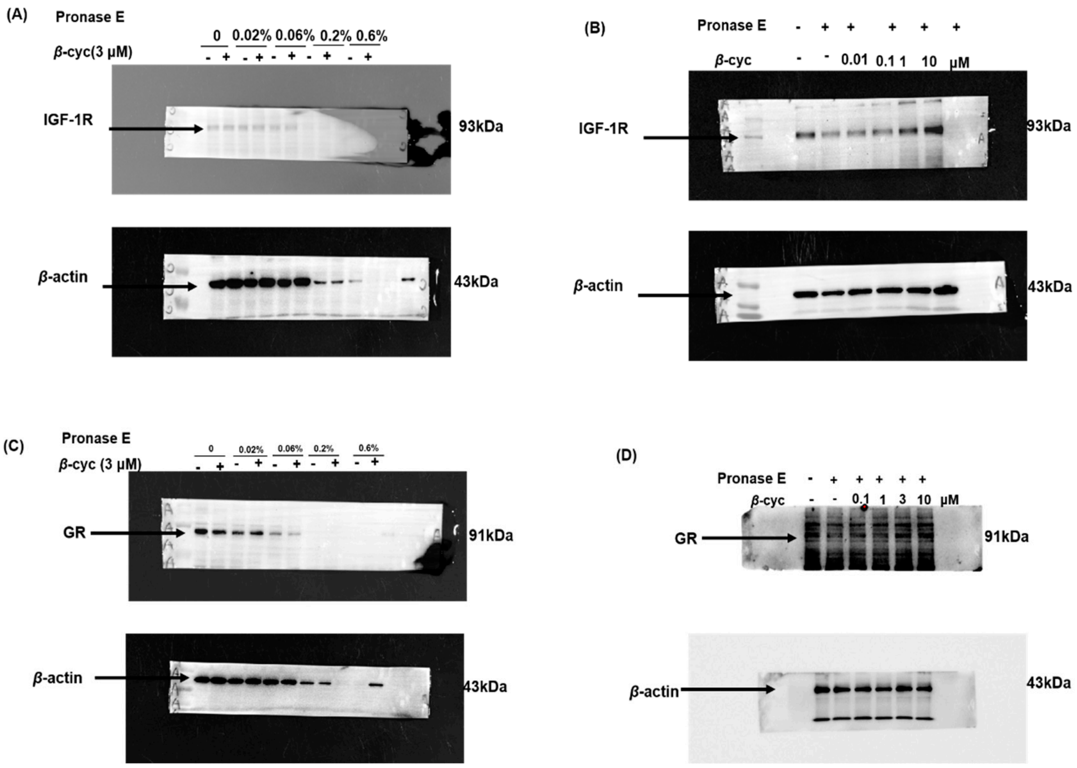

Figure S6: (A-D) Original data of western blot analysis of IGF-1R, GR and *β*-actin in Figure 7A-B and 7D-E.
